# Virtually Accompanied Eating in the Outpatient Therapy of Anorexia Nervosa

**DOI:** 10.3390/nu15173783

**Published:** 2023-08-30

**Authors:** Melina Vogel, Aurora Gil, Camila Galaz, Pascuala Urrejola, Lucas Lacalle, Raúl Jara, Verónica Irribarra, Matias Letelier, Daniela Costa, Gabriela Espinoza

**Affiliations:** 1 Eating Disorders Unit of the UC Christus Health Network, Santiago 8330077, Chile; mvogel@uc.cl (M.V.); agils@ucchristus.cl (A.G.); cgalaza@ucchristus.cl (C.G.); purrejol@med.puc.cl (P.U.); lilacalles@uc.cl (L.L.); rjara@ucchristus.cl (R.J.); virribar@uc.cl (V.I.); meletelier@uc.cl (M.L.); dcosta@ucchristus.cl (D.C.); 2 Psychiatry Department, Faculty of Medicine, Pontificia Universidad Catolica, Santiago 8330077, Chile; 3 Adolescent Unit, Pediatrics Department, Faculty of Medicine, Pontificia Universidad Catolica, Santiago 8330077, Chile; 4 Nutrition and Diabetes Department, Faculty of Medicine, Pontificia Universidad Catolica, Santiago 8330077, Chile

**Keywords:** eating disorders, anorexia nervosa, online meal support groups, weight recovery, geographical barriers

## Abstract

Background: Normalizing the eating pattern and weight recovery are the main objectives in treating anorexia nervosa (AN). Eating accompaniment through shared mealtimes is a common strategy in eating disorder management programs. This study aims to examine the impact on weight gain of an internet-delivered meal support group on patients with AN who were under ambulatory treatment with the Eating Disorders Unit of the UC Christus Health Network, Chile. Methods: An observational study of 54 female patients with AN diagnosis who participated in Online Meal Support Groups (OMSGs) three times a week was performed. Their weight, BMI and BMI%, was reviewed at the beginning of the sessions and at 45- and 90-day follow-up. Results: Patients showed significant weight gain during follow-up. At the 90-day follow-up, patients had gained 4.41 (SD ± 2.82) kg with an effect size of −1.563. Conclusions: Statistically significant differences were found between the weight at the beginning of the intervention and at the 45- and 90-day follow-up, meaning that eating support online groups may be an effective intervention for weight gain and maintenance in patients with AN. These findings highlight the viability of developing cost-effective and more accessible interventions for AN and thus help reduce the duration of untreated disease and its consequences.

## 1. Introduction

Anorexia nervosa (AN) is a mental disorder characterized by an altered body image and intense fear of gaining weight, which leads to behaviors that interfere with maintaining healthy body weight, such as dietary restrictions or purges [1]. AN is a serious illness that significantly impacts an individual’s quality of life, affecting mainly young women [2]. The female-to-male prevalence ratio is around 10:1; however, AN is probably underdiagnosed in males [3]. Between 0.3% and 1% of women will develop AN at some point in their life [4], with a lifetime prevalence of 0.9% among adult females [5]. The incidence rates of AN have been stable in the last decades, although some studies suggest an increase [5], especially in young women [4].

According to the findings available in the literature, AN is associated with significantly increased mortality rates compared to controls selected from the general population [6]. The mortality risk is greatly increased for patients who received in-patient treatment and considerably higher than for other eating disorders (EDs) [7,8]. The standardized mortality ratio for severely malnourished patients with AN can be as high as 15.9 [5]. Due to malnourishment and cognitive and emotional symptoms, AN patients have impairments in attention, processing speed, visual and verbal memory, and visuospatial construction [9,10]. They also have a high rate of anxiety and depressive comorbid disorders [11].

Medical stabilization, nutritional rehabilitation, weight gain, and maintenance are essential pillars in the treatment of AN [12]. Inpatient treatment effectively increases body weight and decreases ED symptoms in patients with low body mass index (BMI) [13]. In hospitalized patients, a weight gain of 0.5–1.4 kg/week is established to avoid the risk of refeeding syndrome, while in outpatients, a weight gain of 250–500 g per week is usually observed [14]. Outpatient, day-hospital-based, or inpatient treatment depends on the ED’s severity but also on access to treatment. Although several treatments can be effective in achieving a healthy weight, if altered eating behaviors (restriction of calories, avoidance of fats, selectiveness, cognitive rigidity towards eating, and food issues, among others) are not dealt with and remain unchanged, relapse risk is increased [15]. Therefore, it is necessary to deal with abnormal eating behaviors as well as weight maintenance.

Healthcare professionals play an essential role in helping AN patients attain healthy body weights and establish normal feeding patterns. This can be accomplished by teaching patients how to replace abnormal eating behaviors with healthy dietary habits [16]. The support and psychoeducation given during mealtimes by nurses, other healthcare professionals, and other patients allow healthy eating patterns to be restituted [17]. Group interventions are also a fundamental part of the treatment since it is known that EDs are diseases with an essential social component in their onset and maintenance [18]. Group engagement can generate a sense of connection usually not found with peers or families during the course of illness [19]. This community experience provides a safe space where those in more advanced stages show their recovery experience to those in earlier stages [20].

All of the above emphasizes the importance of appropriate and timely treatment for AN in order to prevent the long-term physical, psychological, academic, family, and social consequences of this disease. However, it has been stated that adequate treatment requires many health resources, together with multidisciplinary and specialized teams. It is known that the main barriers to patients’ access to treatment are still the availability of skilled health teams and their economic costs [21].

There is growing evidence of the efficacy of internet use in psychological treatment and mental health illnesses [22]. A meta-analysis by Andersson et al. (2014) showed that face-to-face vs. internet-based treatments had similar efficacy [23]. The main advantages of the latter are greater accessibility, lower costs, and flexibility [24]. After the COVID-19 pandemic, healthcare systems worldwide had to adapt to ensure effective treatment for people suffering from AN [25]. The impact of the COVID-19 pandemic on EDs is still being studied. However, some preliminary results show a rise in the number of hospitalizations following the onset of the pandemic, with a 48% increase in hospital admissions compared to a similar period the previous year [26]. As much as 65% of individuals with EDs experienced a worsening of symptoms during the confinement [27]. Disruptions of daily activities due to lockdown, including decreased opportunities for regular food shopping, social interactions, or physical activity, led to the worsening of ED symptoms, such as dietary restraint or binge eating, and higher levels of anxiety and depressive symptoms in ED patients [28,29]. Social distancing requirements or outright bans on in-person interactions changed many social interactions to video-supported communications [30]; for some individuals, exposure to their own image may have increased body image concerns [28].

The effectiveness of online workshops for caregivers of ED patients has also been studied. Family-Based Therapy, based on the Maudsley Model, given by telemedicine platform, has been shown to be effective for both weight recovery and maintenance, thereby improving access for families in remote locations [23]. The Internet-delivered Maudsley Model for adult patients with anorexia also ensured weight gain and fewer worries regarding food [31]. The SUCCEAT program was designed for caregivers of adolescent patients with AN to improve their skills and reduce the caregiver burden, distress, and psychopathology. Truttmann et al. (2020) found a high adherence to this program, with medium to long-lasting effects [32]. In turn, Rodriguez Guarin et al. (2021) analyzed an internet-delivered program in the context of COVID-19 confinement for the treatment of EDs. This program included individual and group interventions and workshops for patients and their caregivers [33]. They found that all respondents considered virtual treatment during confinement feasible and valuable. Pellegrini et al. (2022) looked at the available evidence for virtual ED prevention programs for children, adolescents, and young adults and suggested that virtual workshops can effectively prevent EDs [34].

Since 2020, the Eating Disorders Unit of the UC Christus Health Network (UTAL for its original name: Unidad de Trastornos Alimentarios) has pursued the use of online resources for the management of patients with AN, initially implemented due to the restrictions inherent to the COVID-19 pandemic, but subsequently sustained as a stable way to improve people’s access to effective treatments. The specific objective of this article is to examine the impact of weight gain of an internet-delivered meal support group on patients with AN who were under ambulatory treatment with the UTAL.

## 2. Materials and Methods

### 2.1. Treatment Model

The Eating Disorders Unit of the UC Christus Health Network (UTAL), Santiago, Chile, has a multidisciplinary approach for treating AN patients adapted from the Maudsley-based treatment for EDs [35]. The team has a broad knowledge of EDs and provides nutrition counseling, family-based therapy, and medications (psychiatric treatment). Adolescent medicine specialists and internists are an integral part of the multidisciplinary team. The treatment team’s capacity to work collaboratively in assessment, treatment planning, and treatment review is essential to effective management. With the different components of treatment working collaboratively together, progress in one domain (such as physical or psychological) will enable and support improvement in each of the other domains. Relatives and caregivers are always included from the beginning.

Family interventions include family-based therapy and support groups for caregivers. These provide clinical skills and theoretical understanding for carers. This aims to develop a collaborative approach and therapeutic work that can be supported and enhanced at home. In addition, nutritional education on EDs and psychoeducational activities are included. These workshops are applicable to people of all genders and all ages with various forms of eating disorders.

Nutritional management of AN aims to normalize eating behavior and weight recovery. Oral feeding resumption with solid food as early as possible, avoiding feeding tube placement, and using oral nutrition supplements are also essential parts of the treatment. A behavioral protocol is therefore used to regulate the eating pattern and weight recovery. This includes set meal times, meal timing/duration, correction of abnormal behaviors, as well as providing meals that meet the estimated calorie requirements to ensure weight gain by age and gender. Other aspects of daily life, such as using toilets and showers, physical activity, and social interactions, are also regulated by relatives or caregivers.

All early interventions are directed toward helping patients to recover and maintain weight. More specifically, treatment seeks to identify and intervene in relational patterns that allow eating symptoms to persist while providing family or caregivers with suitable tools to sustain the patient’s improvements. The outpatient setting is preferred at all stages of the treatment unless the patient’s nutritional status or psychiatric comorbidity calls for inpatient management.

### 2.2. Access to Treatment in the UC Christus Health Network Eating Disorder Unit (UTAL)

Most patients admitted to UTAL are outpatients from all over the country. Families come to the unit referred by other mental health teams or professionals or after their relatives notice symptoms suggesting EDs like weight loss, refusal to eat, vomiting, and changes in eating behaviors in general. Patients are admitted into the UTAL after evaluation by the multidisciplinary team. Families are briefed in an interview by a team professional on how the program works, therapies, activities, and costs.

Each family is responsible for paying the expenses of the treatment. Health insurance in Chile is a mixed system with limited coverage for psychiatric and psychological treatments of any kind, including online meal support sessions.

### 2.3. Espacio Balance and Online Meal Support Groups

**Figure A1:**
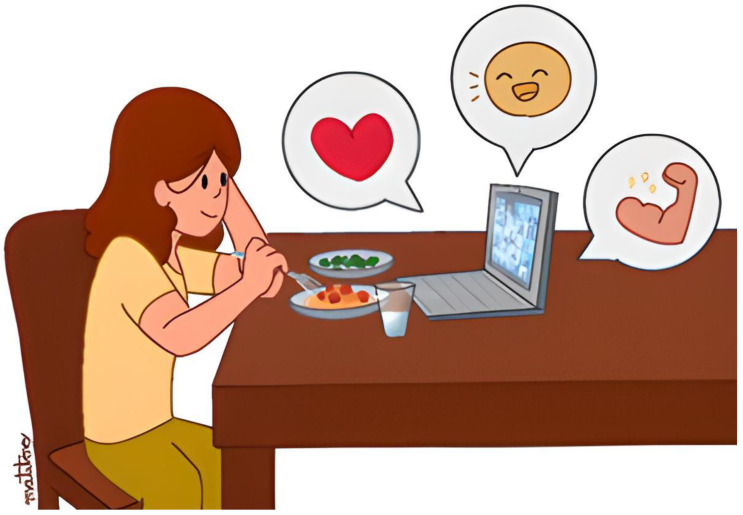
Participant in OMSG interacting with other patients and a member of UTAL who supervises and encourages them through meal time.

Espacio Balance (EB) is a virtual therapy group program designed to support and come into contact with ED patients who need to improve their relationship with food, body perception, self-esteem, and communication with peers (Appendix A Figure A1). These workshops are aimed at treating biased cognitive processing, but at the same time, caregivers are requested to participate in group therapy based on the Maudsley Method [36,37].

Espacio Balance was created during the COVID-19 pandemic at the time of the strictest confinement to facilitate access to treatment for patients who otherwise could not have continued their treatments. This internet-based group has been a valuable tool, especially for patients from more isolated or rural areas where the presence of ED specialists is scarce. Since its development, it has been part of the treatment of ED patients managed at UTAL.

Espacio Balance’s online meal support group (OMSG) was designed to help with nutritional rehabilitation. OMSGs are provided by well-trained professionals, including dietitians, psychiatrists, and psychologists who follow an internal guideline developed to regulate all aspects of social eating. Meetings occur from Monday to Friday at lunchtime. Participants connect via Zoom, and they are supervised by one of the members in an appointment that lasts one hour. The presence in OMSG of well-trained mealtime monitors that belong to the UTAL provides the support and encouragement patients need to work on abnormal eating behaviors and their difficulties. They offer a safe space for emotional containment, where the peer group becomes essential, promoting nutritional restoration, establishing a structured eating plan, and overcoming fear of foods and food-related anxiety.

### 2.4. Access to the OMSG

For admission to the UTAL, patients and their families are interviewed by a psychologist and a psychiatrist. In these interviews, amongst other issues, patients and their caregivers receive a registration packet of written forms and video information about UTAL’s outpatient treatment and the OMSG, which are included in the UTAL treatment approach. In addition, this instance is used to solve questions about the treatment and the online-based groups.

Our registration packet includes a description of the UTAL functioning, consent forms for caregivers and patients, appointment frequency of private practices of the team, cost data of overall treatment, and detailed OMSG information. The patient and their caregivers are asked to complete and sign the forms. Once the registration packet is completed, patients are eligible to register on the OMSG.

Participants of the OMSG are patients who have recently been admitted to the UTAL and are, therefore, at an early stage of treatment. This means they are underweight and likely to be fearful, anxious, and distressed about receiving our help. They are usually ambivalent about weight gain as a therapeutic goal and have difficulties in weight restoration. All patients of the OMSG have a food plan designed by their nutritional team according to their needs. Additionally, to the information given in the admission process, patients receive a written document with detailed information on how the OMSG works before entering. This includes details that should be considered while having lunch at OMSG, such as risks relating to data protection due to using online technology. Acknowledged issues such as internet access, visual impairments, and creating a calm dining atmosphere are essential considerations when starting the OMSG—also, some aspects of correct use of the Zoom platform.

Some crucial points will be explained during the meal, such as the rhythm and times of the meals and a caring atmosphere of respect among the participants. However, issues are highlighted that should not be addressed as they are related to illness, weight, calories, comments from others about weight or appearance, and the amount of food eaten. Furthermore, what to do in case of doubts or difficulties and when to contact your treatment team. All the above points are reinforced during OMSG.

### 2.5. Sample Description

The clinical records of patients with AN of the restrictive subtype diagnosis were reviewed between August 2020, when Espacio Balance started, and December 2022. The inclusion criteria for this study were patients (over 15 years) with AN who were under ambulatory treatment with the UTAL team; patients participated a minimum of three days a week in the OMSG as part of their outpatient treatment for recovery from AN in the Espacio Balance (EB) program.

All the study participants were undergoing treatment at the UTAL. Patients and families who were not willing to accept the commitment required to participate or have a condition that made them unstable, like drug addictions or alcohol abuse, were not eligible. The patients came from different towns in Chile. Body Mass Index (BMI) and Percent Median Body Mass Index (%mBMI) were calculated using WHO growth charts. They were recorded from the dietitians’ files on their first appointment, at the beginning of treatment, and on days 1, 45, and 90 of OMSG’s partaking. Data analysis was performed using the SPSS Version 29 statistical methodology.

## 3. Results

The clinical records of 54 female patients diagnosed with AN were reviewed. The mean age of the group was 18.4 (SD ± 2.6) years. The mean BMI was 16.6 kg/m^2^ (SD ± 1.1 kg/m^2^) with a mean %mBMI of 79.61% (SD ± 6.28%) and weight of 43.1 kg (SD ± 4.6 kg) at the time of admission to the UTAL (see Table 1).

The mean BMI was 17.6 kg/m^2^ (SD ± 1.3 kg/m^2^) with a BMI% 84.2% (SD ± 6.9%) at the beginning of the virtually accompanied eating sessions. The mean BMI was 18.5 kg/m^2^ (SD ± 1.2 kg/m^2^) at the 45-day follow-up, with a BMI% of 88.4% (SD ± 6.9%), and the mean BMI was 19.2 kg/m^2^ (SD ± 1.4 kg/m^2,^) with a BMI% of 9.3% (SD ± 7.4%), at 90 days follow-up (see Table 1).

The mean weight of participants at the time of entry to the OMSG (M = 45.52, SD ± 4.47) was compared with their weight at the 45-day follow-up (M = 48.04, SD ± 4.83). Through a paired sample *t*-test, a *t*(53) = −7.81, *p* < 0.001 was obtained, showing a significant mean weight gain of 2.52 (SD ± 2.37) kg. The sample size effect was calculated with Cohen’s test with a result of −1.064 (see Table 2).

The same comparison and tests were applied to the mean weight at 45 and 90 days of follow-up (M = 49.93, SD ± 4.95), obtaining a result of *t*(53) = −8.31, *p* < 0.001, with a significant weight gain of 1.88 (SD ± 1.66) kg and an effect size of −1.131. Similar analyses were conducted for the mean weight at the beginning of the virtually accompanied eating group sessions and the 90-day follow-up (*t*(53) = −11.48, *p* < 0.001), showing a significant weight gain of 4.41 (SD ± 2.82) kg and an effect size of −1.563.

## 4. Discussion

Refeeding is one of the most challenging aspects of AN treatment. Feeding failure is often the main indication for hospitalization, with consequent financial costs, disruption of routines, academic life, and family stress [38]. The availability of support programs for both families and patients has a vital role in managing EDs [39,40,41], especially in societies where access to treatment by specialized teams is scarce and expensive, with low coverage of mental health services.

Online platforms’ use increased in importance during the time of social confinement during the COVID-19 pandemic [42]. Since then, they have become part of the ED’s treatment options [32]. The benefits of using them include lowering costs and allowing wider access to mental health care for ED patients. In the least developed countries, specialized ED teams are often centralized in large cities, leaving whole geographical areas without the presence of specialists. This results in long traveling times for patients and their families and hence a barrier to treatment access. This is particularly important in a country with geographical singularities, such as Chile, which extends over more than 4000 km. In addition, despite the high demand in Chile and the fact that intensive treatments, such as day hospitals, are a widely accepted therapeutic resource and have been present worldwide for some 20 years, in Chile, there are only a few centers dedicated to such an approach.

Using an internet-based approach during the COVID-19 pandemic also enabled ED patients to access peer support groups during social isolation, generating a sense of belonging with less stigma or shame. In the three years of running the virtual support platform (Espacio Balance), we learned how important it is for patients and their relatives to have a secure space where they can meet other people with similar issues and share their experiences and learning.

A long duration of untreated illness is an adverse prognostic factor in AN and is associated with chronic illness progression [43,44]. The use of OMSG not only enables us to begin treatment but also provides mental health care for patients in their initial phases of the disease. It may contribute to a more favorable illness course and improved prognosis. The most critical factors influencing treatment initiation have been described as emotional and practical support given by primary healthcare professionals and close relatives [45]. OMSG and Espacio Balance provide both practical support and treatment tools that may resemble these factors, which often need to be improved in these patients.

The refeeding process is necessary for undernourished patients with AN and can be challenging since they must gain weight without developing refeeding syndrome. Even though this is less often described in outpatients, difficulties are still encountered. As AN is a psychiatric disorder, patients always fear gaining weight. Nutritional rehabilitation is the most critical part of treatment and involves recovery of dietary habits that have been intentionally avoided. Restoration of body weight allows to correct many of the physical complications of malnutrition and improves patients’ motivation to collaborate in the treatment. Espacio Balance treatment groups provide nutrition education as part of the treatment of ED and play a crucial role in helping patients to change their food attitudes and routines, lose fears, and recover a healthy eating pattern. All these changes may contribute to improving their nutritional status. Caregivers can accompany patients when they start OMSG. This allows them to learn from the OMSG therapist how to attend to their ill relatives during meal times. This may also contribute to the nutritional recovery.

To the authors’ knowledge, this is the first experience in Latin America with online supportive eating therapy. At OMSG, with the guidance of a therapist and a protocol of expected behaviors during eating, as well as peer support, patients are encouraged to identify the main symptoms of AN, their interpersonal and behavioral consequences, and how they disrupt their social life. Espacio Balance gives positive messages regarding the importance of a well-balanced diet and the benefits of treatment. It also offers tools to fight the intense fear associated with refeeding and the importance of family and community as support during treatment.

In this work, we wanted to show the positive impact of OMSG in an outpatient setting in the context of a multidisciplinary treatment of AN. This study explored weight gain and weight maintenance in patients with AN who participated in online group mealtime support sessions. Statistically significant differences were found between weight at baseline and at 45- and 90-day follow-up, which may suggest that OMSG is an effective intervention for weight gain and maintenance in patients with AN. In our experience, OMSG patients, adequately guided by trained therapists, begin functioning as a protective community. Many of them stated in their individual therapy that they had not had similar previous experiences but rather the opposite, having focused on social media that promoted strict diets, extreme thinness, or bodybuilding, where the path to social acceptance was to submit to rigid aesthetic canons and unbalanced eating behaviors. This impression is widely replicated in other expert ED teams and observed in the recent review by Dane and Bathia [46]. OMSG can contribute to correcting erroneous beliefs and influencing young subjects through a different community experience, emphasizing the normalization of eating behavior and amplifying health and self-care messages.

This research may open the possibility of new studies to scrutinize other variables involved in weight regain in patients with anorexia who participate in online group interventions, such as OMSG.

This study was conducted using observational and descriptive methodology. An essential limitation of the study was the lack of a control group, considering the ethical barriers of having a group of patients without an intervention that seemed urgent and necessary. The small number of patients included could be explained by the exceptional circumstances when this intervention was developed during lockdown for the COVID-19 pandemic and by the nature of the disease. Patients and families joining the Espacio Balance therapy groups and the OMSG were previously interviewed by members of the therapy team, which could incorporate a potential selection bias. Most patients affected by AN are female, as is the sample in this study. This could be a limitation to extrapolate our results to other groups of patients. Nevertheless, this intervention could be an effective way of providing access to treatment for patients who live far away from highly specialized treatment centers, in rural areas, or have limited access to mental health care.

## Figures and Tables

**Table 1:** Descriptive characteristics of the sample.

	*n*	Minimum	Maximum	Mean (SD)
Age (years)	54	14	26	18.39 (2.58)
UTAL initial weight	53	32.80	53.90	43.12 (4.59)
UTAL initial BMI	54	12.42	18.29	16.59 (1.10)
UTAL initial %mBMI	54	61.49%	97.29%	79.61% (6.28%)
OMSG initial weight	54	36.10	58	45.52 (4.48)
OMSG initial BMI	54	14.91	21.32	17.57 (1.27)
OMSG initial %mBMI	54	70.49%	102.60%	84.18% (6.91%)
OMSG weight 45 days	54	36.80	61.90	48.04 (4.83)
OMSG BMI 45 days	54	16.14	22.46	18.52 (1.19)
OMSG %mBMI 45 days	54	76.49%	106.14%	88.35% (6.93%)
OMSG weight 90 days	54	37.80	64.50	49.92 (4.95)
OMSG BMI 90 days	54	16.80	24.35	19.23 (1.39)
OMSG %mBMI 90 days	54	79.23%	114.86%	91.34% (7.38%)

**Table 2:** Paired samples *t*-test for initial and evolutional average weight in EB.

		Mean (SD)	Std Error	95% Confidence Interval of Difference Lower/Upper	*t*	*df*
Pair 1	OMSG entry weight—OMSG weight 45 days	−2.52 (2.37)	0.32	−3.17/−1.87	−7.81 *	53
Pair 2	OMSG weight 45 days—OMSG weight 90 days	−1.88 (1.66)	0.22	−2.33/−1.43	−8.31 *	53
Pair 3	OMSG weight entry—OMSG weight 90 days	−4.41 (2.82)	0.38	−5.17/−3.63	−11.48 *	53

UTAL: Eating Disorders Unit of the UC Christus Health Network (UTAL for its original name: Unidad de Trastornos Alimentarios). OMSG: online meal support group. * One-sided and two-sided *p* < 0.001.

## Data Availability

Raw data were generated at the UC Eating Disorders Unit. Derived data supporting the findings of this study are available from the corresponding author upon request.
